# Editorial: Mechanisms and practices for the management of plant-soil biota interaction

**DOI:** 10.3389/fpls.2024.1399420

**Published:** 2024-04-09

**Authors:** Sergio Saia, Emanuele Radicetti, Katharina Pawlowski, Sabine Dagmar Zimmermann, Andrea Genre

**Affiliations:** ^1^ Department of Veterinary Science, University of Pisa, Pisa, Italy; ^2^ Department of Chemical, Pharmaceutical and Agricultural Sciences, University of Ferrara, Ferrara, Italy; ^3^ Department of Ecology, Environment and Plant Science, Stockholm University, Stockholm, Sweden; ^4^ IPSiM, CNRS, INRAE, Institut Agro, Univ Montpellier, Montpellier, France; ^5^ Department of Life Science and Systems Biology, University of Turin, Turin, Italy

**Keywords:** plant-soil biota management, ecosystem, agro-ecosystem, genotypes, beneficial fungi, viruses, bacteria

The perception of the importance of the soil biota for agricultural production, land management, and ecosystem management and functioning increased in the last decades. Plenty of local, national and international initiatives and research projects are addressing the relationship between plant and soil biota, using both holistic metabarcoding/metagenomics-based studies and mechanistic approaches addressing bipartite plant-microbe interactions. In most of these studies attention has been focussed on associations and symbioses, which may simultaneously promote plant health, crop yield, microbial spread, and microbiota-associated ecosystem services, with special emphasis on soil health and preservation. Indeed, the soil microbiota is the main component in soil fertility and can directly influence major soil traits at both the short and the long term. So far, the management of the soil biota, especially for those components that improve crop production, is scarcely explored even in studies that aim at introducing sustainable cropping systems and natural soil management. In particular, the benefits deriving from individual microbial taxa have been demonstrated under diverse conditions, but scientific literature is far from providing general guidelines to achieve the strongest benefits from the management of soil microbiota.

This Research Topic (RT) was launched in January 2023 on the trail of two previous RTs, namely ***Arbuscular Mycorrhizal Fungi: The Bridge between Plants, Soils, and Humans*** and ***Insights in Plant Symbiotic Interactions: 2021***, both deepening insight in beneficial plant-symbiotic interactions and aiming to elucidate the functioning and applicative potential of such interactions. The present RT focussed on the management of these and other symbioses for improving plant growth, soil traits and food safety for both livestock and humans. The call for papers attracted several contributions, among which 7 original papers and 1 perspective article were accepted (**Table 1**).

**Table 1:** Summary of the focus and approaches presented in each contribution of the RT.

Paper	Microbes	Metabolism	Plant Genus	-omics
Mun et al.	PGPR bacteria (*Bacillus*)	N	*Oryza* *Glycine*	yes
Wang et al.	Bacteria (multiple)	C, N, P	*Casuarina*	yes
Fu et al.	Bacteria (multiple) Fungi (multiple)		*Zea*	
Fan et al.	Bacteria (multiple)	P	*Populus*	
Koczorski et al.	Bacteria (P-solubilizing)	P	*Salix*	yes
Mortier et al.	Fungi (arbuscular mycorrhizal)	P	*Juglans* *Zea*	
Qiao et al.	Fungi (multiple)		*Paeonia*	yes
Li and Gao	Bacteria (multiple) Fungi (multiple)			

Phosphorus is one of the most essential elements for plant growth and development. However, phosphorus is also one of the hardest nutrients for plants to get because of its low availability in the soil due to the rapid formation of poorly soluble complexes (Bindraban et al., 2020). Koczorski et al. have investigated the effect of phosphate-solubilizing bacteria inoculation on the development and transcriptomic scenario of two willow species under different phosphate concentrations. Regarding agricultural practices, Fan et al. studied phosphorus availability for growth of seedlings from the endangered species *Populus euphratic*a (Ling et al., 2015). The authors analysed within a field experiment with two-year old seedlings the effect of cow dung return methods on soil microbiota and phosphorus within the root zone. In addition to soil properties, abundance of soil bacteria and genes involved in phosphorus solubilization and transformation have been analysed. Findings showing that use of biochar (carbonised cow manure; Kurniawan et al., 2023) increased the biodiversity of the microbial community as well as the availability of phosphorus indicate clearly a way to reduce phosphate fertilisation thus improving agricultural management.

Also related to phosphate nutrition (Plassard et al., 2019; Zhang et al., 2024), but in a rather different context, Mortier et al. present the results of their study of a simulated agroforestry system, where they demonstrated the role of a common mycorrhizal network (Walder et al., 2015) in phosphate distribution between intercropped walnuts and maize plants. Fungal communities have been studied also in the context of cultivation of a rare and endangered plant species in China, the shrub *Paeonia ludlowii*. A previous study described the rhizosphere microbial diversity and community structure in the introduction area (Gao et al., 2022). Here, growth behaviour of wild and cultivated species was compared and related to rhizosphere soil fungi (Qiao et al.). Network analyses revealed that Ascomycota were the key fungi in wild species but were absent in the cultivated ones. The authors concluded that a disturbed soil fungi community promoting pathogenic fungi might hinder the cultivation of the studied plant species. Similar network analyses for domesticated plants have already shown such negative plant-soil feedback (Carrillo et al., 2019; Martín-Robles et al., 2020; Chang et al., 2021). These results point clearly to the fact that the introduction of plant species to another environment can cause a shifted soil-microbiome-plant equilibrium and must be taken into account for plant cultivation.

According to the above-cited studies, plant roots throughout the release of different exudates, *i.e.* low-molecular weight organic acids and metabolites, and their interaction with water and nutrient cycles contribute to the modification of soil physicochemical characteristics determining a selective environment for the microorganisms’ establishment and development (Kuzyakov and Razavi, 2019). It is necessary to develop models able to enhance our understanding of the plant-driving microbial community distribution patterns in rhizospheric soil. Fu et al. suggest that the evaluation of the genetic structure of the soil microbiome of the plant rhizosphere could be adopted for the evaluation of these aspects. In particular, the study of Fu et al. aims to examine the role of microdiversity in influencing the distribution of rhizosphere-associated microbial species across environmental gradients from root surface to bulk soil at the operational taxonomic units (OTUs) and amplicon sequence variant (ASVs) levels. The finding suggests that ASVs exhibited microdiversity within OTUs in the root-associated soil compartments of mature maize plants growing under field conditions. In addition, environmental factors in root-associated soil compartments of maize may drive the microdiversity in microbial community structure.

Another key topic in this RT is the interaction between soil microbes and cultivated plants. Under this approach, the adoption of plant growth-promoting rhizobacteria (PGPR) may be of interest for their benefits on the agro-physiological characteristics of crops. The study of Mun et al. aims at the evaluation of rhizobacterium *Bacillus aryabhattai* on soybean and rice. The findings showed how *B. aryabhattai* affects amino acids and protein metabolism in terms of quantity and quality, thus indicating *B. aryabhattai* as a promising PGPR for field application not only for soybean and rice but also for various other crops.

Different groups focused on the assembly of rhizosphere microbiomes and their development over time. In 2001, Batish et al. (2001) had concluded that *Casuarina equisetifolia*, a tree with high resistance against abiotic stress and important in coast line protection, had allelopathic properties in that phenolics, in particular, 2,4-di-tert-butylphenol (2,4-DTBP), released from the photosynthetic branchlets and litter of the tree adversely affected the understorey vegetation. Recently, Xu et al. (2022) suggested that actually, litter fungi with higher abundance in young and mature plantations were involved in the synthesis of 2,4-DTBP. Based on rhizobiome metagenomics, Wang et al. conclude that a decrease of actinobacteria from 10 key genera led to reduced metabolic capacity of the soil microbiome for nutrient cycling together with reduced ion exchange capacity of the rhizosphere soil. This result offers options for ameliorating the negative effects of continuous planting of *C. equisetifolia* that not only affect the understorey vegetation but also *C. equisetifolia* itself. The conclusions also fit the hypothesis that host filtering effects are the main deterministic force in the assembly of microbiota, a process otherwise controlled by stochastic processes. However, Li and Gao, focussing on the root endophytome, propose in their intriguing *Perspective* article that dispersal limitation represents a further deterministic process, in particular with regard to the bacterial microbiome, which has to be considered when attempting microbiome engineering.

In summary, the majority of the studies included in this RT applied -omic techniques to investigate the relationship between bacteria and fungi and different crops. For agricultural applications, it will now be interesting to delve deeper into mechanisms and best practices for managing plant-soil biota on the field-scale, with more attention on whole microbiome interactions and herbaceous crops, in particular legumes and C3 cereals.

## Author contributions

SS: Writing – original draft, Writing – review & editing. ER: Writing – original draft, Writing – review & editing. KP: Writing – original draft, Writing – review & editing. SZ: Writing – original draft, Writing – review & editing. AG: Writing – original draft, Writing – review & editing.
